# A Circo-Like Virus Isolated from *Penaeus monodon* Shrimps

**DOI:** 10.1128/genomeA.01172-13

**Published:** 2014-01-16

**Authors:** Hanh T. Pham, Qian Yu, Maude Boisvert, Hanh T. Van, Max Bergoin, Peter Tijssen

**Affiliations:** a INRS, Institut Armand-Frappier, Laval, QC, Canada; b Institute of Tropical Biology, Vietnam Academy of Science and Technology, Ho Chi Minh City, Vietnam

## Abstract

A virus with a circular Rep-encoding single-stranded DNA (ssDNA) (CRESS-DNA) genome (PmCV-1) was isolated from *Penaeus monodon* shrimps in Vietnam. The gene structure of the 1,777-nucleotide (nt) genome was similar to that of circoviruses and cycloviruses, but the nucleic acid and protein sequence identities to these viruses were very low.

## GENOME ANNOUNCEMENT

Recently, viral metagenomics revealed circo-like viruses in the marine copepod species *Acartia tonsa* and *Labidocera aestiva* (Crustaceae) (1). Here, we report the isolation by classical methods of a similar virus with a circular Rep-encoding single-stranded DNA (ssDNA) (CRESS-DNA) genome from *Penaeus monodon* shrimps (PmCV-1).

Circoviruses are nonenveloped, icosahedral particles and contain circular ssDNA genomes of about 1.7 to 2.3 kb. The open reading frame (ORF) for the Rep protein codes for conserved rolling circle replication (RCR) and superfamily 3 (SF3) helicase motifs (2, 3). In contrast, the *cap* gene is generally not conserved. Originally, circoviruses were isolated from pig and bird species (4–6), but *in vitro* rolling circle amplification, high-throughput sequencing, and metagenomics studies have led to rapid expansion of the known diversity and host range of small CRESS-DNA viruses (CVs). This also led to an unsettled viral taxonomy with different subfamilies within the *Circoviridae* family and reassignment of their members (2).

In this study, about 100 g of cleaned, diseased *Penaeus monodon* shrimps was homogenized and virus was purified (isolate VN11 from Vietnam) as described previously (7, 8). Viral DNA was isolated from purified viruses with the High Pure viral nucleic acid kit (Roche Applied Science), followed by rolling circle amplification by ø29 DNA polymerase (NEB) at 30°C for 6 hours (9). Amplified product was then digested with EcoRI and separated on a 1% agarose gel. A band of 1.8 kb was recovered from the gel and cloned into a pBluescriptKS(+) vector. Clones were sequenced by Sanger’s method and primer walking. PCR with outward primers was carried out and the amplicon was cloned into a TA vector (pGEMT-easy, Promega). All sequencing results were assembled using the CAP3 program (10).

Sequence analysis revealed that PmCV-1 is closely related to members of the *Cyclovirus* genus in the *Circoviridae* family. PmCV-1 possesses a 1,777-nucleotide (nt) genome containing three ORFs encoding 266, 255, and 146 amino acids (aa). Numbering starts with the loop in the conserved stem loop. The 266-aa product of the largest ORF, from nt 51 to 851, shared about 30% sequence identity (over 90% of query coverage) with the putative Rep of cycloviruses and contained RCR and SF3 motifs. The 255-aa product of the ORF translated in the opposite direction, from nt 1,671 to 904, shared 25% identity with the Cap protein of a *Diporeia* sp.-associated circular virus (GenBank accession no. KC248415.1, *E* value 0.004), and thus the ORF probably encodes the capsid protein. The smallest ORF, from nt 1,246 to 1,686, codes for a 146-aa protein that did not reveal any amino acid similarity using Blastx in a protein database with *E* values of <0.01. The 156-nt intergenic region between the 5′ ends of putative *cap* and *rep* genes encompasses 13-nt inverted repeats (nt 11 to 23 and 1765 to 1777) forming a stem and a 10-nt loop containing a canonical nonanucleotide, TAATATTAC, between nt 2 and 10. The intergenic region between the 3′ ends of the *cap* and *rep* genes is 53 nt long. The genome structure resembles that of circoviruses and cycloviruses.

Metagenomic discovery has particularly impacted the discovery of CRESS-DNA viruses, both in host range and genetic diversity. Although this approach is very powerful, its perils should not be underestimated (11).

### Nucleotide sequence accession number.

The GenBank accession number for PmCV-1 is KF481961.
